# Evolution of energy utilization during metamorphosis in rapid developing *Drosophila melanogaster*

**DOI:** 10.17912/micropub.biology.001654

**Published:** 2025-07-14

**Authors:** Abhishek Kumar Farand, Adwait Mishra, Neha Rauhila, Surbhi Yadav, Mallikarjun Shakarad

**Affiliations:** 1 Department of Zoology, University of Delhi, New Delhi, Delhi, India

## Abstract

Maintenance of energy homeostasis is essential for survival. Reduced development time is often associated with tradeoffs among life history traits due to limited time in acquiring energy reserves. Late third instar larvae of *Drosophila melanogaster* selected for faster development and increased longevity (FLJs) had triacylglycerol comparable to their ancestral control (JBs) populations, despite their reduced larval duration. However, FLJ adults have non-significantly reduced lipid levels at emergence and significantly reduced starvation resistance post-emergence, indicating altered energy utilization during pupal stage. These findings suggest that the cost of rapid development arises not from energy acquisition, but from its utilization and deployment during metamorphosis.

**Figure 1. Effect of selection on metabolic phenotypes:**
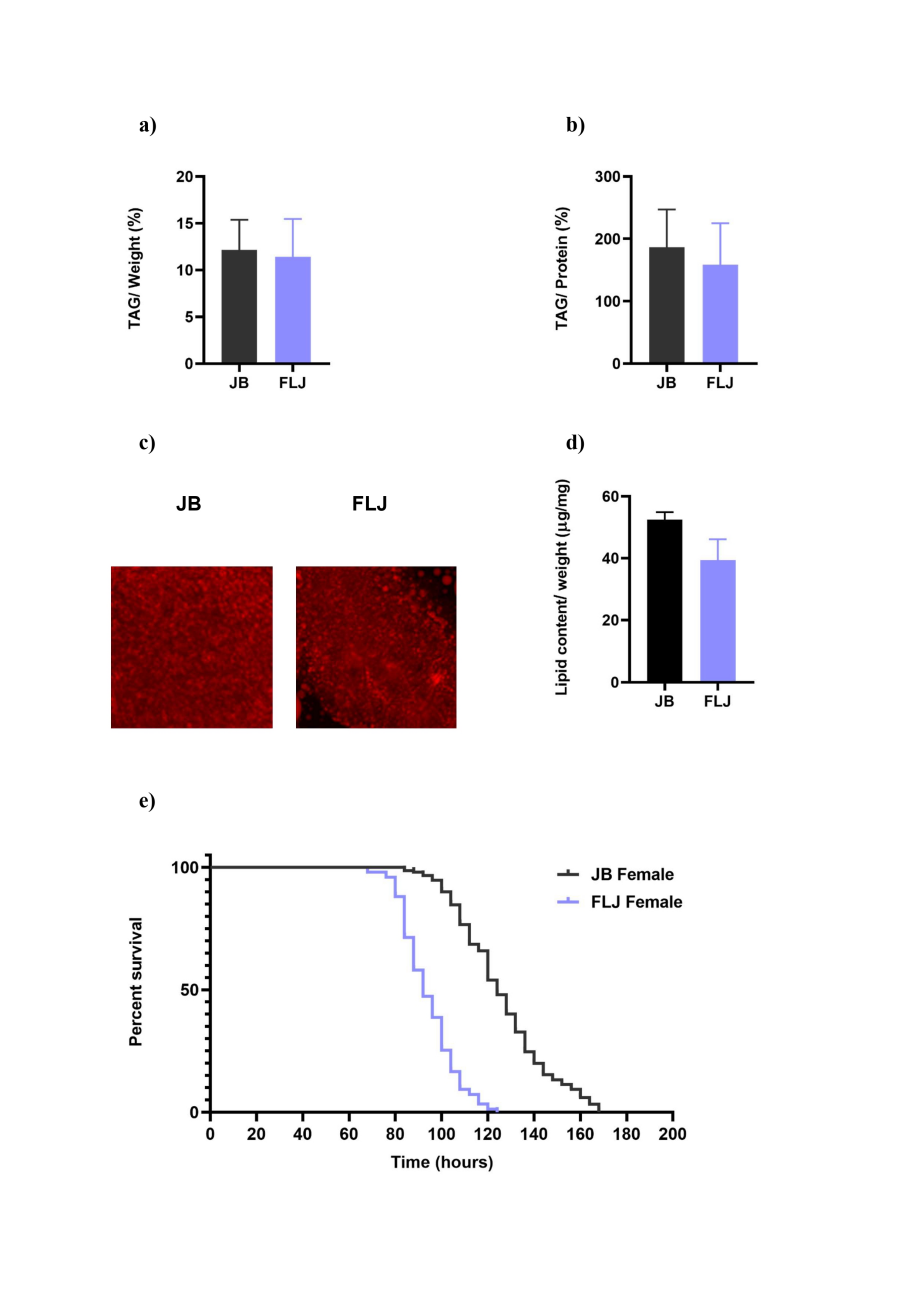
(a) Triglyceride/ weight was comparable between control (JBs) and selected (FLJs). (b) TAG/ protein was comparable between JBs and FLJs. (c) Lipid droplets accumulation in larval abdominal fat body was comparable. (d) Total lipid levels/ weight were non-significantly reduced in FLJs at emergence. (e) Percent survival during starvation was significantly reduced in FLJs. For TAG, data is represented in percentage normalized by either weight (a) or protein levels (b). Data for a, b and d are represented as mean ± SEM.

## Description

Maintenance of energy homeostasis- balance between energy intake, storage and utilization is remarkable capability of living organisms. Nutrients acquired during feeding enter metabolic pathways leading to synthesis of biomolecules that ultimately generate energy. The biomolecules that are in excess are stored in specialized tissues and are utilized during phases of limited food availability and during functions requiring high energy like immune response against infection (Zhao et al., 2022). In *Drosophila* , fat body serves as a dynamic tissue to control the storage and utilization of fat to meet the energy demands of the fly throughout its developmental stages (Arrese & Soulages, 2010). It is also involved in innate immunity, detoxification, regulating insect development and behavior by sensing various nutritional and hormonal signaling and by releasing various fat body regulatory signals (Buchon et al., 2014; Canavoso et al., 2001; Lemaitre & Hoffmann, 2007; Li et al., 2019). It contains intracellular organelles known as lipid droplets (LDs) which serve as a storehouse of triacylglycerols (TAGs), main component of stored lipids. During energy requirements or situations of energy depletion, lipases access the TAGs and hydrolyze them to produce glycerols and fatty acids that serve as a major source of energy (Arrese & Soulages, 2010; Blumrich et al., 2021).

In *Drosophila melanogaster* , larval duration is a critical window during which flies accumulate energetic reserves primarily in the form of triacylglycerides (TAGs) that are essential for completing metamorphosis and supporting survival during early adult life (Carvalho et al., 2012; Heier & Kühnlein, 2018). Experimental evolution studies have reported different tradeoffs associated with reduced development time including, reduced adult size, energy storage, stress resistance and fitness parameters, with many of these tradeoffs believed to arise from reduced resource accumulation during the larval stages (Chippindale et al., 1997; Handa et al., 2014; Nunney, 1996; Prasad et al., 2001; Shakarad et al., 2005; Sharma & Shakarad, 2021). In this study, we used *Drosophila melanogaster* populations simultaneously selected for (i) faster pre-adult development and (ii) increased longevity (FLJs), and their ancestral control populations (JBs). The selected FLJs have significantly reduced development time (~7.5 days) compared to control JBs (~9.5 days). The reduction in development time is primarily due to reduction in larval duration, with no corresponding reduction in pupal duration (Sharma et al., 2020). We hypothesized that FLJs, due to their reduced development time, would accumulate fewer resources (TAGs) during the larval stage and consequently exhibit lower starvation resistance at emergence as TAGs stores are essential reservoir for surviving during starvation (Bi et al., 2012; Grönke et al., 2003, 2007; Gutierrez et al., 2007).

Interestingly, selected FLJs exhibit comparable whole body TAG levels either normalized by weight (F _1, 4_ = 0.020, *p=0.894* ; Figure 1a) or by protein levels (F _1, 4_ = 0.096, *p=0.771* ; Figure 1b) during late third instar larval stage. The size of lipid droplets in the abdominal fat body is also similar to that of JBs (Figure 1c). This suggests that, despite their reduced larval feeding duration, FLJs accumulate resources at levels equivalent to JBs by the time they exit the feeding stage, just prior to pupation. Therefore, FLJs ensure sufficient energy reserves that would be utilized during tissue reorganization during the non-feeding but metabolically active pupal stage. The comparable larval feeding rate of the selected FLJs with that of control JBs (Sharma et al., 2020) and similar lipid droplets in the fat body (this study, Figure 1c), suggest similar nutrient acquisition and storage in the FLJ and JB populations. However, at the time of eclosion, the lipid levels per unit weight were non-significantly reduced in FLJs compared to JBs (F _1,4_ = 3.271, *p* = *0.144* ; Figure 1d). Further, the FLJ adults exhibit significantly reduced starvation resistance compared to their JB counterparts (chi square= 186.2, *p<0.0001* ; Figure 1e). The reduced lipid levels at emergence and reduced starvation resistance immediately post-emergence in the FLJs suggest that relatively higher energy is being utilized for tissue reorganization during the pupal stage. This highlights that the tradeoffs associated with rapid development emerges not from energy acquisition, but from its utilization and deployment during metamorphosis.

## Methods

***Drosophila* maintenance protocol:**

A total of six *Drosophila melanogaster* populations were used in this study. Three populations were simultaneously selected for faster pre-adult development and increased longevity (FLJs) and three were their ancestral control populations (JBs). The ancestry and detailed maintenance protocol has been published in many studies (Chandrashekara & Shakarad, 2011; Chauhan et al., 2020; Handa et al., 2014; Sharma et al., 2020; Sharma & Shakarad, 2021; Shrivastava & Shakarad, 2023). Briefly, both populations’ cultures were maintained at standard laboratory conditions of 25 ± 1 ºC temperature, 70 ± 5% relative humidity, 24:0::L:D cycle in Powers Scientific Inc. USA, environmental chambers and reared on a standard Banana–Jaggery media (SM). JBs have 21 days egg to egg discrete generation cycle in which eggs were collected in 40 glass vials per replicate population with ~6 ml SM and all eclosed flies were transferred to cages on 12 ^th^ day from the egg collection day (ECD). In FLJs, eggs were collected in 80 glass vials per replicate population with ~6ml SM and all flies that eclosed within ~7.5 days post egg collection were transferred to cages (selection for faster pre- adult development). Two sister cages were maintained to prevent adult overcrowding. When approximately 50% mortality was observed in either cage, eggs were collected to initiate the next generation (selection for increased longevity). At the time of initiation of this study, JBs and FLJs had been through 456 and 232 generations cycles, respectively. Before initiating each experiment, JBs and FLJs were subjected to identical rearing conditions for one generation to eliminate any non-genetic parental effects. Eggs were collected in 40 glass vials with 6ml SM and emerging adults were transferred to plexi glass cages on 10 ^th^ day from ECD for FLJs and 12 ^th^ day from ECD for JBs. The assay larvae were obtained from these flies and egg collection was staggered based on the development time difference. This study was performed on late L3 female larvae and female virgin adults.

**Triglyceride storage:**

Whole body triglyceride content was estimated by following the manufacturer’s protocol of Triglyceride (TG) Colorimetric Assay Kit GPO-PAP Method (Make-Elabscience, Catalogue No.- E-BC-K238). Briefly, 10 larvae were weighed and homogenized in 300μl of 0.1M PBS. Samples were centrifuged at 12000 rpm for 10 minutes at 4 ⁰C. 7 μl of supernatant of sample and Glycerinum standard solution were plated in duplicates on 96 well plate. Then, 250μl of working solution was added to each well followed by incubation at 37 ⁰C for 10 minutes in dark. Absorbance was measured at 510nm using ELISA plate reader. Two samples per replicate population of control and selected were analyzed.

**Protein estimation:**

Whole body protein content was estimated by following the manufacturer’s protocol of Pierce ^tm^ BCA Protein Assay Kit (REF-23227). To estimate protein content, 25μl of supernatant was taken from the same sample used for Triglyceride content quantification. Standard of Bovine serum albumin and samples were plated on 96 well plate and 200μl of working reagent (50 BCA reagent A: 1 BCA reagent B) was added to each well. 96 well plate was shaken for 30 seconds and incubated for 30 minutes at 37 ⁰C. After incubation, absorbance was measured at 562 nm using ELISA plate reader.

**Lipid droplet staining:**

Lipid droplets were stained following the protocol of (Aditi et al., 2016) with slight modifications. Fat bodies were dissected out from the female larvae in ice cold 1X PBS. Then, these tissues were fixed in 4% formaldehyde in 0.1% Triton X-100 for 20 minutes. After fixation, tissues were rinsed with 1X PBS thrice for 10 minutes each. Tissues were placed in 0.5mg/ml solution of Nile red diluted in PBS for 30 minutes. Subsequently, tissues were again rinsed thrice with 1X PBS for 10 minutes each and mounted in Vectashield mounting medium. Tissues were examined under Zeiss fluorescent microscope.

**Starvation assay:**

Freshly eclosed adult female flies within 2 hours of eclosion were used for starvation sensitivity assay. 10 flies were transferred to glass vials containing non-nutritive agar (1.24%). Every vial was observed at an interval of 4 hours to assess mortality until the death of all flies. The mid-point of two 4 hour interval checks was taken as the time until death. The total time duration from set-up time till death in hours for each fly was taken as the absolute starvation resistance.

**Total lipids estimation:**

5 samples (10 female flies per sample) per replicate population were used to estimate the lipid content by following the protocol of (Handa et al., 2014). Flies were dried in oven at 70 °C for 36 hours to obtain dry weight. Then, flies were transferred to 1.5 ml micro centrifuged tube containing 1 ml of diethyl ether at room temperature on a gel rocker and lipids were extracted for 48 hours with three ether changes at an interval of 12 hours. Flies were again dried for 2 hours at 35 °C to obtain lipid free weights. Difference between dry weight and lipid free weight was considered as the lipid content.

**Statistical analysis:**

Shapiro-Wilk normality test and Bartlett's test of homogeneity were used to assess the nature of data. TAG/ weight, TAG/ protein, and total lipids/ weight data were normally distributed. Thus, one way ANOVA was used for statistical analysis with selection as fixed factor and replicate blocks as random factor. Survival probabilities of JBs and FLJs were assessed using non-parametric Kaplan Meier analysis followed by log-rank (Mantel-Cox) test.
